# Assessment of coronary endothelial function using blood oxygenation level dependant cardiovascular magnetic resonance imaging (BOLD-CMR) in a canine model

**DOI:** 10.1186/1532-429X-13-S1-P53

**Published:** 2011-02-02

**Authors:** Flewitt A Jacqueline, Matthias Vöhringer, Jordin D Green, Todd Anderson, John V Tyberg, Matthias G Friedrich

**Affiliations:** 1 Stephenson CMR Centre / University of Calgary, Calgary, AB, Canada; 2 Robert-Bosch-Krankenhaus, Stuttgart, Germany; 3 Siemens Healthcare, Calgary, AB, Canada; 4 Libin Cardiovascular Institute of Alberta/University of Calgary, Calgary, AB, Canada

## Objective

To test whether BOLD-CMR can detect endothelium-dependent coronary vasodilatation.

## Background

Endothelial function is important in the pathogenesis of atherosclerosis. Whereas endothelial function of peripheral circulation can be assessed non-invasively with Flow-Mediated Dilatation, no method exists for coronary circulation. BOLD-CMR utilizes inherent contrast, where the signal intensity (SI) is linearly correlated with the regional tissue level of deoxygenated hemoglobin. This technique may have potential to assess coronary endothelial function non-invasively.

## Methods

In 6 anesthetized dogs, after instrumentation of the left circumflex coronary (LCx) artery with a coronary infusion catheter and an extravascular flow probe, the coronary endothelium was activated with acetylcholine (ACh). Using a clinical 1.5-T MRI (MAGNETOM Avanto, Siemens Healthcare, Erlangen, Germany) mid short axis cine SSFP BOLD sequence (as previously described^1^) was performed at baseline and during graded ACh infusion in the LCX (0.1 μg/min, 1.0 μg/min and 10 μg/min). Scan parameters were: field-of-view 228 x 280 mm; matrix size 125 x 192; in-plane resolution 1.8 x 1.6 mm; slice thickness 5 mm; TR/TE 5.8 ms/2.9 ms; flip angle 90°; readout bandwidth 275 Hz/Px; signal averages 1; typical breath-hold 16 seconds. CMR imaging was repeated with 10 µg ACh after pharmacological blockage of endothelial NO-synthetase by Nω-nitro-L-arginine methyl ester (L-NAME). Coronary flow, coronary sinus (CS) oxygen saturation (SvO2) and BOLD-CMR imaging were performed simultaneously. Images were analyzed using clinically validated software (cmr^42^, Circle cardiovascular imaging, Calgary Canada).

## Results

**Figure 1:**
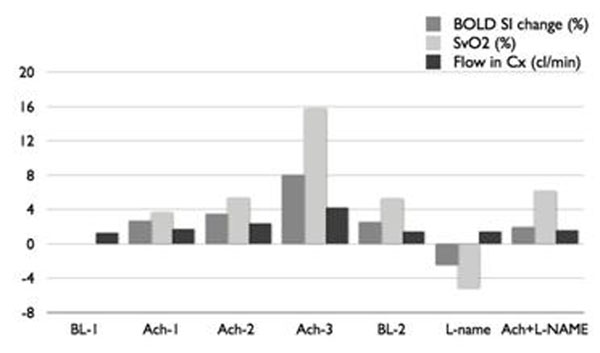
BOLD SI and CS SvO_2_ percent change from baseline. LCx Flow (cl/min)

Pharmacological interventions and image acquisition was successful in all dogs. Compared to baseline there was a significant increase in LCX flow at each ACh infusion grade (28%, 86%, 210%), which was accompanied by significant increases in CS SvO2 (3.6%, 5.3%, 15.7%) and in BOLD-CMR SI (means and confidence intervals) (2.7%, 0 to 5.8%, p=0.05; 3.5%, 0.4 to 7.0%, p<0.05; 8.0%, 4.2 to 11.3%, p<0.01). Compared to baseline, after L-NAME administration, flow response to the highest level of ACh was blunted (20%) and no significant increase of BOLD-CMR signal intensity was found (1.9%). Figure 1.

## Conclusions

BOLD-sensitive CMR allows for non-invasive imaging of changes in myocardial oxygenation induced by endothelium-dependent vasodilatation. Further studies should address the utility of BOLD-CMR in the clinical setting of endothelial function testing.
